# Molecular Characterization and Phylogenetic Analysis of Subgroup III Bovine Respiratory Syncytial Virus from a Dairy Outbreak in Thailand

**DOI:** 10.3390/vetsci13030220

**Published:** 2026-02-26

**Authors:** Preeda Lertwatcharasarakul, Sakuna Phatthanakunanan, Jaturong Wongsanit, Porawit Saisanongyod, Ploypassorn Homklinkaew, Suwimon Phandee

**Affiliations:** 1 Department of Pathology, Faculty of Veterinary Medicine, Kasetsart University, Kamphaengsaen Campus, Kamphaengsaen, Nakhon Pathom 73140, Thailand; preeda.le@ku.th (P.L.);; 2 Kamphaengsaen Veterinary Diagnostic Center, Faculty of Veterinary Medicine, Kasetsart University, Kamphaengsaen, Nakhon Pathom 73140, Thailand; sakuna.p@ku.th; 3 Department of Large Animal and Wildlife Clinical Science, Faculty of Veterinary Medicine, Kasetsart University, Kamphaengsaen Campus, Kamphaengsaen, Nakhon Pathom 73140, Thailand; jaturong.w@ku.th; 4 Kasetsart University Veterinary Teaching Hospital Nong Pho, Faculty of Veterinary Medicine, Kasetsart University, Potharam, Rachaburi 70120, Thailand; porawit.s@ku.th

**Keywords:** bovine respiratory syncytial virus, subgroup III, phylogenetic analysis, *G* gene, dairy cattle, Thailand

## Abstract

Bovine respiratory syncytial virus (BRSV) is a major viral pathogen associated with bovine respiratory disease (BRD), a leading cause of illness and economic loss in cattle worldwide. In June 2022, an acute respiratory outbreak occurred in a dairy herd in Photharam District, Ratchaburi Province, Thailand, affecting 25 of 103 cows (24.3%) and resulting in three deaths (2.9%). This study aimed to confirm BRSV as the etiological agent of the outbreak and to genetically and phylogenetically characterize Thai BRSV strains using partial G gene sequencing. Clinical signs included fever, nasal discharge, coughing, and subcutaneous emphysema. Nested reverse transcription-polymerase chain reaction (nested RT-PCR), a sensitive method for detecting viral RNA and targeting the *F* and *G* genes, confirmed BRSV in all samples. At the same time, bovine parainfluenza virus type 3 (BPIV-3), bovine viral diarrhea virus (BVDV), and bovine herpesvirus 1 (BoHV-1) were not detected. Phylogenetic analysis of partial *G* gene sequences showed that all Thai isolates clustered closely within subgroup III, with 100% nucleotide identity among themselves and 85.9–97.7% similarity to subgroup III strains from other countries. Amino acid alignment indicated conservation of key antigenic motifs, including the cysteine noose, with only minor substitutions compared to some foreign strains. This study provides the first genetic and phylogenetic characterization of BRSV in Thailand, highlighting the genetic stability of subgroup III and providing baseline molecular data to support regional surveillance, diagnostics, and vaccine strategies.

## Simple Summary

Bovine respiratory syncytial virus (BRSV) is a significant pathogen contributing to the bovine respiratory disease complex, a major cause of morbidity and economic losses in both dairy and beef cattle worldwide. In Thailand, previous research on BRSV has been mainly limited to seroprevalence studies. This report identifies Subgroup III of BRSV as the causative agent of a respiratory disease outbreak in a dairy farm in Photharam District, Ratchaburi Province. It is the first molecular detection of BRSV associated with a respiratory disease outbreak in dairy cattle in Thailand. Understanding the genetic and phylogenetic characterization of BRSV is crucial for developing effective biosecurity measures and vaccination strategies.

## 1. Introduction

Bovine respiratory disease (BRD) poses a major health and economic challenge in cattle industries worldwide, resulting in significant financial losses due to reduced weight gain, lower productivity, and increased management, treatment, and prevention costs, particularly in affected calves [1,2]. BRD is a multifactorial syndrome caused by a complex array of viral and bacterial pathogens, including bovine herpesvirus 1 (BoHV-1), bovine respiratory syncytial virus (BRSV), bovine parainfluenza 3 virus (BPIV-3), bovine viral diarrhea virus (BVDV), *Mycoplasma bovis*, *Pasteurella multocida*, *Mannheimia haemolytica*, and *Histophilus somni* [3,4,5,6,7]. Despite extensive research and control efforts, BRD remains a primary clinical concern in cattle [3,5,6].

Among the viral agents involved in BRD, BRSV is a key pathogen that often causes severe respiratory illness, especially in young calves and feedlot cattle [3,8,9,10,11]. BRSV infection weakens the host’s respiratory defenses, making it easier for secondary bacterial infections to occur and increasing disease severity [5,12]. Due to the significant impact of BRSV on cattle health and productivity, understanding its molecular characteristics and evolutionary relationships is crucial for developing effective control and prevention methods [13,14,15,16,17].

BRSV is an enveloped, negative-sense, single-stranded RNA virus. It belongs to the genus Orthopneumovirus (formerly Pneumovirus) in the family *Pneumoviridae*. Its genome is about 13.4–15.1 kb long. BRSV encodes ten mRNAs that produce eleven proteins: fusion (F) glycoprotein, attachment (G) glycoprotein, small hydrophobic (SH) protein, nucleocapsid (N) protein, polymerase (L), phosphoprotein (P), matrix (M) protein, two matrix accessory proteins (M2-1 and M2-2), and nonstructural proteins (NS1 and NS2). The viral envelope displays three surface glycoproteins F, G, and SH, which are essential for host cell entry and immune evasion [18,19]. The G glycoprotein mediates viral attachment and acts as a major antigenic determinant. The F glycoprotein promotes membrane fusion and contributes to viral pathogenicity [17,19,20]. These surface proteins are key targets of the bovine immune response and show notable variability among field isolates.

Monitoring the genetic diversity and phylogenetic relationships of BRSV is crucial for guiding biosecurity efforts and for developing vaccines targeting locally circulating strains [17,21]. BRSV exhibits significant genetic variability, resulting in antigenic diversity and potentially leading to vaccine failure in the field [17]. Consequently, molecular epidemiological studies have focused on key immunogenic genes, particularly *G*, *N*, and *F*, to investigate viral evolution and transmission. Notably, the *G* gene is frequently used for classifying viral subgroups, and specific nucleotide variations in the *G* gene have been linked to differences in clinical outcomes [12,15,16,22,23,24].

Worldwide, phylogenetic analyses show BRSV isolates fall into multiple genetic lineages, often matching geographic origin [25]. Initially, seven distinct subgroups (I–VII) were defined by *G* gene sequences [13,26]. Later studies, including work on Japanese strains, expanded this to ten subgroups (I–X) [23]. These groups differ in antigenic traits and global distribution, highlighting the need for ongoing molecular surveillance to monitor new variants [13,15,23,24]. For example, subgroup III viruses are found in North America, Europe, and Asia [13,23]. Phylogenetic comparisons clarify relationships between modern field strains and historical reference strains and offer insights into BRSV’s evolutionary dynamics [9,16,21].

In Thailand, most BRSV research has focused on serological surveys of cattle herds, with only a few molecular detection studies conducted to date [27,28]. A recent RT-PCR survey detected BRSV RNA in 52.6% of cattle respiratory samples, confirming the virus’s circulation [28]. However, no reports have been published on the genetic characterization or phylogenetic subgrouping of BRSV in Thai cattle. Therefore, the objective of this study was to confirm BRSV as the detected viral agent and to genetically and phylogenetically characterize the outbreak strains based on partial G gene sequencing and to identify the circulating subgroup.

## 2. Materials and Methods

### 2.1. Specimen Collection

In June 2022, an outbreak of acute respiratory disease occurred on a commercial dairy farm in Photharam District, Ratchaburi Province, Thailand (Supplementary Figure S1), housing 103 lactating cows. A total of 25 cows (24.3%) exhibited respiratory signs, and three deaths (2.9%) were recorded during the outbreak. Because all affected animals originated from the same farm and showed similar clinical signs, clinical specimens were collected from a subset of affected animals for laboratory investigation.

Four clinical specimens were collected by a local veterinarian, including one lung tissue sample obtained post-mortem from a 5-year-old cow and three nasal swabs collected from symptomatic cows aged 3 to 8 years (Supplementary Table S1). Nasal samples were collected using sterile cotton swabs and immediately placed into sterile phosphate-buffered saline (PBS). Lung tissue was collected within approximately 2 h after death using sterile procedures, placed in a sterile specimen bag, and transported on ice to the laboratory for further analysis.

### 2.2. Study Design and Specimen Selection

The present study was conducted as an outbreak investigation focused on preliminary molecular characterization of the detected virus rather than population-level statistical inference. Subsampling for molecular analysis was based on specimen availability and sample quality at the time of investigation. One lung tissue sample from a fatal case was included to increase the chances of detecting viral nucleic acid from the lower respiratory tract, while nasal swab samples from three clinically affected animals were selected to represent actively symptomatic cases within the herd. Due to the limited number of suitable specimens available during the outbreak investigation, formal statistical sampling methods were not applied. Accordingly, the selected specimens were not intended to be statistically representative of the entire herd but were used to provide preliminary molecular and phylogenetic information on the detected BRSV strain.

### 2.3. Nucleic Acid Extraction

Total nucleic acids were extracted from each sample using the IndiMag Pathogen Kit (Indical Bioscience, Leipzig, Germany; Cat. No. SP947457) following the manufacturer’s instructions. In brief, 200 µL of homogenized tissues or PBS wash of nasal swabs were added into the first column (lysate) and extracted by the IndiMag 48 automated system (Indical Bioscience, Leipzig, Germany); 100 µL of extracted nucleic acids were obtained from the fourth column (elution slot) (Supplementary S1). They were aliquoted and stored at −80 °C. No prior quantification was performed because standard protocols were used, and extracts were used directly for RT-PCR analyses.

### 2.4. PCR and RT-PCR Detection

Complementary DNA (cDNA) was synthesized from 5 µL of extracted nucleic acids in a total reaction volume of 25 µL using the RevertAid First Strand cDNA Synthesis Kit (Thermo Fisher Scientific, Vilnius, Lithuania; Cat. No. K1622) with random hexamer primers. Subsequently, 2 µL of cDNA was used to detect BRSV, BPIV-3, and BVDV by RT-PCR using published primer sets [16,28,29]. For the detection of BoHV-1, 2 µL of extracted nucleic acid was used directly as the DNA template in a PCR reaction with a total volume of 25 µL [30]. All amplification reactions were prepared using DreamTaq Green PCR Master Mix (Thermo Fisher Scientific, Lithuania; Cat. No. K1082) according to the manufacturer’s instructions, and cycling parameters were applied as described in the respective publications. Amplicons were separated by electrophoresis on 1.5% agarose gels stained with RedSafe™ Nucleic Acid Staining Solution (iNtRON Biotechnology, Gyeonggi, Republic of Korea; Cat. No. 21141)

Amplicons of the first round of nested RT-PCR (1030 bp) of BRSV-positive *G* gene samples were purified and sequenced using the Illumina MiSeq platform (BTSeq™ Contiguous Sequencing Service, CELEMICS, Seoul, Republic of Korea). Partial *G* gene sequences obtained in this study were deposited in GenBank under accession numbers OQ799973–OQ799976.

### 2.5. Sequencing Analysis and Phylogenetic Construction

Initially, consensus nucleotide sequences were compared with the GenBank database using the BLAST program (https://blast.ncbi.nlm.nih.gov/Blast.cgi, accessed on 15 January 2025) to confirm viral identity. The partial *G* gene sequences obtained in this study, together with reference isolates retrieved from the GenBank database (https://www.ncbi.nlm.nih.gov/genbank/, accessed on 15 January 2025) (Table 1), were aligned using ClustalW implemented in MEGA version 11 (https://www.megasoftware.net/, accessed on 24 June 2022) [31]. Genetic distances were estimated with the Kimura two-parameter model, and phylogenetic relationships were inferred using the neighbor-joining method with 1000 bootstrap replicates to assess nodal support. All sequence alignments, distance calculations, and tree reconstructions were performed in MEGA version 11 [31].

## 3. Results

A commercial dairy herd of 103 lactating cows in Ratchaburi Province, located in western Thailand (Supplementary Figure S1), experienced an acute respiratory disease outbreak. Twenty-five animals (24.3%) showed clinical signs, including fever, nasal discharge, and coughing. Three cows died (2.9% overall mortality), resulting in a case fatality rate of 12% among symptomatic animals. The outbreak lasted approximately two weeks, with most new cases occurring during the first seven days. The affected cows that died showed acute clinical signs and succumbed to the disease within 1–2 days after the onset of respiratory symptoms. Supportive treatment, including fluid therapy, antimicrobial drugs and non-steroidal anti-inflammatory drugs (NSAIDs), was administered at the farm level; however, no antiviral treatment was available, and the affected animals did not respond to therapy. Gross pathological examination of lung tissue collected post-mortem revealed multifocal dark red consolidation, interlobular edema and emphysema, and features of interstitial pneumonia, consistent with a viral respiratory infection.

Four clinical specimens were examined, including one post-mortem lung tissue sample from a cow and three nasal swabs from clinically affected cows. All four samples tested positive for BRSV by nested RT-PCR using both *F* and *G* gene primer sets. Amplicons of the expected sizes (833 bp for the *F* gene and 541 bp for the *G* gene) using nested RT-PCR were detected in all samples. The *G* gene amplicons from the first PCR round were purified and sequenced for further analysis. No samples were positive for BPIV-3, BVDV, or BoHV-1.

**Figure 1:**
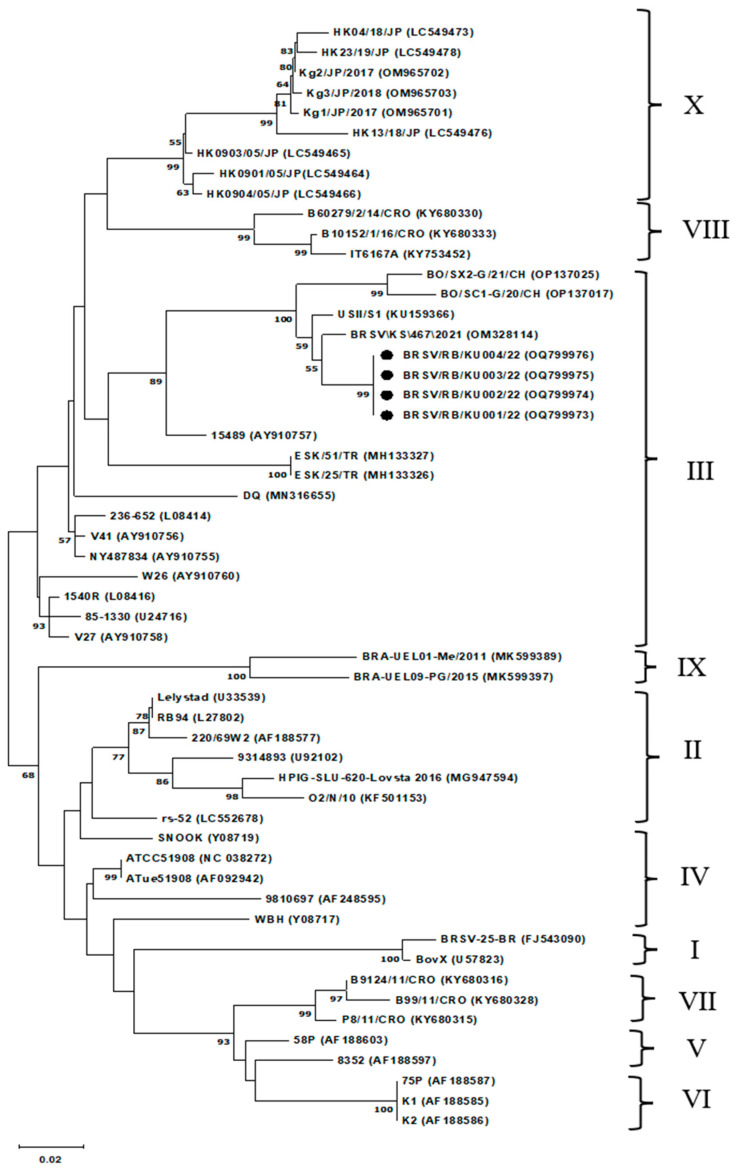
Phylogenetic tree constructed using partial *G* gene sequences of BRSV and reference strains retrieved from the GenBank database. The tree was generated using the Neighbor-Joining method, with bootstrap support values (1000 replicates) indicated above the branches. Branch lengths are drawn to scale, with the units representing distances used to infer the phylogenetic relationships. The Roman numerals (I–X) indicate the phylogenetic subgroups of BRSV based on partial *G* gene sequence analysis. Thai isolates are marked with black dots.

Phylogenetic analysis of partial *G* gene sequences (Figure 1) revealed that all four Thai isolates clustered within subgroup III of BRSV. The neighbor-joining tree with 1000 bootstrap replicates provided strong support (>90%) for this grouping. The Thai isolates were genetically identical to each other. They shared 97.7% and 97.4% nucleotide similarity with BRSV/KS/467/2021, isolated from the USA in 2021 (GenBank accession no. OM328114), and USII/S1, isolated from the USA in 2015 (KU159366), respectively. Overall similarity with other subgroup III strains from the United States, China, Turkey, and Italy ranged from 85.9% to 97.7%. In contrast, sequence similarity with subgroups I, II, and IV–X ranged from 79.0% to 86.7% (Table 2), confirming the genetic distinctness of subgroup III and its role in the Ratchaburi outbreak.

**Figure 2:**
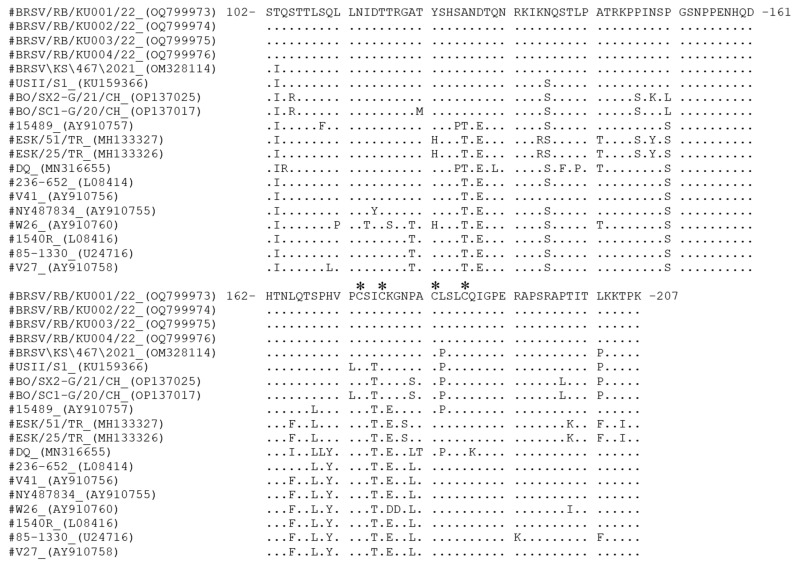
Amino acid sequence alignment of residues 102–207 of the G protein from subgroup III of Thai isolates and BRSV genotype III isolates deposited in the GenBank database. Dots indicate identical amino acid residues compared to the first row of sequences (BRSV/RB/KU001/22). The positions of amino acid residues are referenced to the DQ strain (GenBank accession no. OM328114). The symbol “#” indicates the strain names included in the alignment, and “*” denotes conserved cysteine residues.

Alignment of deduced amino acid sequences (residues 102–207 of the G protein) showed complete conservation among the four Thai isolates (Figure 2). In contrast, international subgroup III strains harbored several substitutions absent in the Thai sequences. Compared to reference strains, the Thai isolates differed by only three residues from BRSV/KS/467/2021 (OM328114) and by six residues from USII/S1 (KU159366). Notably, substitutions L183P (in both comparisons), as well as P172L and I175T (in USII/S1), were located within the central conserved region (residues 158–189).

Four cysteine residues (C173, C176, C182, and C186) were conserved in all isolates, preserving the structural integrity of the G protein. The sequences also showed a high frequency of serine (S) and threonine (T) residues, consistent with predicted glycosylation sites. Notably, three residues associated with antibody reactivity, including P180, L183, and S184, appeared in the Thai isolates.

## 4. Discussion

BRSV remains a leading cause of bovine respiratory disease (BRD) in many countries, including Thailand [4,14,15,23,26,28,32,33]. Serological evidence indicated a herd prevalence of 71.6% in dairy cattle across 31 provinces of Thailand in 1995 using an ELISA test [27]. More recently, molecular detection revealed that 52.6% of nasal swab samples collected from 152 calves with respiratory signs in Chiang Mai Province (2002–2021) were positive for BRSV by real-time RT-PCR [28]. The present study documents an acute BRD outbreak at a dairy farm in western Thailand (Ratchaburi Province), affecting 24.3% of the herd, with a mortality rate of 2.9% and a case fatality rate of 12%. Together, these findings address the study objective by confirming BRSV as the detected viral agent in this outbreak and by identifying the circulating phylogenetic subgroup (subgroup III) based on partial G gene sequencing.

RT-PCR was performed to detect BRSV, BPIV-3, and BVDV, while conventional PCR was used to detect BoHV-1. These viruses are recognized causes of BRD in cattle [3,4,5,6]. Only BRSV was identified as the causative agent in this outbreak, reinforcing its role as a primary pathogen within the bovine respiratory disease complex (BRDC). Subsequent sequencing and phylogenetic analysis of the partial *G* gene revealed that all Thai isolates clustered tightly within subgroup III, exhibiting 100% nucleotide identity among themselves, consistent with clonal spread within the herd. The lack of significant divergence suggests the outbreak may have originated from a single introduction or an undetected persisting lineage. Moreover, the Thai isolates shared 85.9–97.7% similarity with subgroup III strains reported in the United States, China, Turkey, and Italy [13,15,23]. The nucleotide similarity observed between Thai isolates and international subgroup III strains indicates close genetic relatedness within this subgroup. In contrast, greater sequence divergence was observed relative to strains from other subgroups, supporting the phylogenetic classification obtained in this study. Although formal statistical comparison of genetic distances was not performed due to the limited number of sequences analyzed, the observed pattern of high intra-subgroup similarity and increased divergence between subgroups is consistent with previously described molecular epidemiological patterns of BRSV evolution [21,23,26].

These findings are consistent with previous reports describing relatively conserved genetic characteristics within BRSV subgroups, while maintaining detectable divergence between subgroups [17,20]. This evolutionary pattern is consistent with previous studies indicating that BRSV evolution is characterized by gradual genetic drift within subgroups rather than rapid lineage replacement, resulting in geographically distributed but genetically related strains [17,20,23]. Similar subgroup-specific clustering patterns and limited intra-subgroup variability have also been reported in BRSV outbreaks in Europe and Asia [13,23,26,34], supporting the concept that regional circulation of genetically related subgroup III strains may occur over extended periods. Nevertheless, continued molecular surveillance is required to monitor potential genetic changes that may influence viral evolution and epidemiological patterns in the region. These findings establish important baseline molecular data for Thailand and support the need for continued surveillance and improved control strategies. Although the involvement of other viral or bacterial pathogens cannot be completely excluded, the consistent detection of BRSV in all examined samples, together with the absence of BPIV-3, BVDV, and BoHV-1, supports BRSV as the primary detected viral agent associated with this outbreak.

The G protein of BRSV is one of the main viral proteins that is used to identify subgroup-specific antigenic differences. The G proteins were separated into portions according to location and function of the protein, including the cytoplasmic portion (residues 1–37), transmembrane regions (residues 38–66), and extracellular portion (remaining amino acid residues) [35]. Thai isolates from this study were compared with amino acid residues 102–207 of the G protein, which is located in the extracellular portion. They identified identity sequences, but several amino acids in the Thai isolates differed from those in foreign subgroup III strains, including a central conserved region (residues 158–189).

The central conserved region is an important immunodominant region that exhibits a characteristic globular structure with two cysteine bridges (C173–C186 external and C176–C182 internal) forming the “cysteine noose” structure [17,36]. Thai isolates were closely related to isolates from the USA (strain BRSV/KS/467/2021 and USII/S1), which differed by 1 and 3 amino acid residues, respectively, from the central conserved region. The cysteine noose was identical among Thai and other subgroup III isolates. Moreover, both USA strains differed at amino acid 183, indicating that the mutation at this position likely has significant structural consequences [37]. Continuous monitoring remains necessary to identify future antigenic changes that could impact diagnostic accuracy or vaccine performance.

Notably, the amino acid substitutions identified in the Thai isolates, including L183P and P172L, were located within the central conserved region of the G glycoprotein, a region known to play an important role in immune recognition and virus–host interaction [17,36]. Previous studies have suggested that amino acid variations within this region may influence antigenic properties and antibody binding [17,20,24,38]; however, the biological significance of these substitutions remains unclear. At present, no functional data are available to determine whether these changes affect viral pathogenicity or immune escape. Therefore, although the Thai isolates clustered within Subgroup III, further experimental studies are required to determine whether these substitutions affect virulence or potentially influence cross-protection conferred by currently available vaccines.

Twenty-three mAbs that recognize seven antigenic regions on the G protein of BRSV can be used to classify the virus into two main lineages [38]. The viruses have been classified into four antigenic subgroups (A, B, intermediate, and untype) based on the reactivity of monoclonal antibodies [37,39,40]. Amino acid sequences at positions 180–184 of the G protein were LACLS, PACSP, and LACSS, which were determined to be typical of antigenic subgroups A, B, and intermediate, respectively. In comparison, Thai isolates were PACLS, identical to isolates from Turkey with ESK/51/TR (GenBank accession no. MH133327) and ESK/25/TR (GenBank accession no. MH133326) and differed from previous antigenic subgroups.

The detection of subgroup III BRSV in Thailand carries important implications for disease management. Expanded molecular surveillance is needed to determine the prevalence and distribution of subgroup III, as well as other subgroups, nationwide. Integration of molecular data with serological surveillance could provide deeper insights into transmission dynamics in both dairy and beef production systems, facilitating earlier detection and improved outbreak response.

Bovine respiratory disease is recognized as a multifactorial syndrome in which viral infections frequently predispose animals to secondary bacterial infections that contribute to disease severity. Although the present study excluded major viral pathogens commonly associated with BRD, including BPIV-3, BVDV, and BoHV-1, bacterial pathogens such as *Mannheimia haemolytica* and *Mycoplasma bovis* were not investigated. The potential contribution of bacterial coinfections, therefore, cannot be excluded. Future studies incorporating both viral and bacterial diagnostics would provide a more comprehensive understanding of BRD outbreaks and disease dynamics in dairy cattle in Thailand.

This study provides baseline molecular and phylogenetic data on BRSV associated with a respiratory disease outbreak in Thailand; these data were sufficient to identify BRSV as the detected viral agent and to determine its phylogenetic subgroup in this outbreak. A limitation of this study is that only a small number of clinical specimens were available for sequencing, and only partial G gene sequences were analyzed. Future studies that include a larger number of BRSV-positive samples and broader genomic coverage would help better assess the genetic diversity and circulation patterns of BRSV in Thailand.

In addition, serological data were not available during the outbreak investigation due to limited access to BRSV serological diagnostic facilities in Thailand at the time of the study. Future investigations integrating molecular and serological approaches, together with expanded diagnostic capacity, would further enhance understanding of BRSV epidemiology in Thailand. Building on this foundation, large-scale molecular and epidemiological surveillance across diverse dairy populations would be valuable for elucidating viral evolution, transmission dynamics, and the effectiveness of intervention and control strategies.

## 5. Conclusions

This study provides the first molecular and phylogenetic characterization of BRSV associated with an acute respiratory outbreak in a dairy herd in Ratchaburi, Thailand, and supplies baseline data to support regional surveillance. The outbreak strains clustered within subgroup III and showed 100% nucleotide identity, with high similarity to international subgroup III strains and conserved antigenic motifs, including the cysteine noose. The observed genetic stability suggests that current molecular diagnostics and subgroup III–based vaccines are likely to remain applicable in Thailand. Future studies should include a larger number of BRSV-positive samples and broader genomic analyses (e.g., full-genome sequencing) to better assess the genetic diversity and circulation patterns of subgroup III BRSV in Thai dairy herds and to inform long-term control strategies.:

## Figures and Tables

**Table 1:** Information on BRSV isolates retrieved from the GenBank database. This table summarizes the details of BRSV isolates used for comparison in this study, including their subgroup classification, year of isolation, country of origin, and GenBank accession numbers. The isolates are grouped by their phylogenetic subgroup (I–X), with information on the geographic distribution and year of isolation for each sequence included in the analysis.

Strain	Subgroup	Year	Country	GenBank Accession No.
BovX	I	1967	Switzerland	U57823
BRSV-25-BR	I	1993	Brazil	FJ543090
rs-52	II	-	-	LC552678
RB94	II	1969	Belgium	L27802
Lelystad	II	1974	The Netherlands	U33539
220/69W2	II	-	Belgium	AF188577
9314893	II	1993	Denmark	U92102
HPIG-SLU-620-Lovsta_2016	II	2016	Sweden	MG947594
O2/N/100	II	2010	Norway	KF501153
BRSV/RB/KU001/22	III	2022	Thailand	OQ799973
BRSV/RB/KU002/22	III	2022	Thailand	OQ799974
BRSV/RB/KU003/22	III	2022	Thailand	OQ799975
BRSV/RB/KU004/22	III	2022	Thailand	OQ799976
236–653	III		USA	L08414
BRSV\KS\467\2021	III	2021	USA	OM328114
USII/S1	III	2015	USA	KU159366
BO/SC1-G/20/CH	III	2020	China	OP137017
BO/SX2-G/21/CH	III	2021	China	OP137025
ESK/51/TR	III	2016	Turky	MH133327
ESK/25/TR	III	2016	Turky	MH133326
1540R	III	-	USA	L08416
DQ	III	2018	China	MN316655
85–1330	III	1985	USA	U24716
V27	III	1986	USA	AY910758
V41	III	1988	USA	AY910756
NY487834	III	-	USA	AY910755
15489	III	-	USA	AY910757
W26	III	-	USA	AY910760
SNOOK	IV	1976	UK	Y08719
WBH	IV	1986	The Netherlands	Y08717
9810697	IV	-	-	AF248595
ATCC 51908	IV	1975	USA	NC038272
ATue51908	IV	1998	Germany	AF092942
58P	V	1998	France	AF188603
8352	V	1998	France	AF188597
75P	VI	1998	France	AF188587
K1	VI	1997	France	AF188585
K2	VI	1997	France	AF188586
B9124/11/CRO	VII	2012	Croatia	KY680316
B99/11/CRO	VII	2012	Croatia	KY680328
P8/11/CRO	VII	2012	Croatia	KY680315
B10152/1/16/CRO	VIII	2016	Croatia	KY680333
IT6167A	VIII	2014	Italy	KY753452
B60279/2/14/CRO	VIII	2016	Croatia	KY680330
BRA-UEL01-Me/2011	IX	2011	Brazil	MK599389
BRA-UEL09-PG/2015	IX	2015	Brazil	MK599397
HK0901/05/JP	X	2005	Japan	LC549464
HK0904/05/JP	X	2005	Japan	LC549466
HK0903/05/JP	X	2005	Japan	LC549465
HK13/18/JP	X	2018	Japan	LC549476
Kg1/JP/2017	X	2017	Japan	OM965701
Kg3/JP/2018	X	2018	Japan	OM965703
Kg2/JP/2017	X	2017	Japan	OM965702
HK04/18/JP	X	2018	Japan	LC549473
HK23/19/JP	X	2019	Japan	LC549478

**Table 2:** Percent similarity of nucleotide sequences of BRSV isolates from this study within subgroup III and between sub-genotypes, based on partial *G* gene sequences.

Subgroup	Within Group	Percent Similarity									
		Between Sub-Genotypes									
		I	II	III	IV	V	VI	VII	VIII	IX	X
Thai isolates	100	80.38–82.08	80.90–84.65	85.92–97.72	81.11–85.03	83.02–83.84	82.53–82.53	78.98–81.24	83.79–85.21	80.96–81.47	82.08–86.70

## Data Availability

The original contributions presented in this study are included in the article/Supplementary Material. Further inquiries can be directed to the corresponding author.

## Supplementary Materials

The following supporting information can be downloaded at: https://www.mdpi.com/article/10.3390/vetsci13030220/s1, Figure S1: Figure of Thailand map showing Ratchaburi Province in dark gray; Table S1: Clinical and animal information of specimens used for molecular detection and phylogenetic anal-ysis of bovine respiratory syncytial virus (BRSV); Supplementary S1: The DNA extraction protocol of IndiMag Pathogen Kit by using IndiMag 48.

## References

[1] Blakebrough-Hall C., McMeniman J.P., González L.A. (2020). An evaluation of the economic effects of bovine respiratory disease on animal performance, carcass traits, and economic outcomes in feedlot cattle defined using four BRD diagnosis methods. J. Anim. Sci..

[2] Overton M.W. (2020). Economics of respiratory disease in dairy replacement heifers. Anim. Health Res. Rev..

[3] Makoschey B., Berge A.C. (2021). Review on bovine respiratory syncytial virus and bovine parainfluenza—Usual suspects in bovine respiratory disease—A narrative review. BMC Vet. Res..

[4] Pardon B., De Bleecker K., Dewulf J., Callens J., Boyen F., Catry B., Deprez P. (2011). Prevalence of respiratory pathogens in diseased, non-vaccinated, routinely medicated veal calves. Vet. Rec..

[5] Potter T., Barrett D., Cutler K., Hart K., Biggs A. (2017). Clinical forum: Bovine respiratory disease. Livestock.

[6] O’Donoghue S., Waters S.M., Morris D.W., Earley B. (2025). A comprehensive review: Bovine respiratory disease, current insights into epidemiology, diagnostic challenges, and vaccination. Vet. Sci..

[7] Barroso-Arévalo S., Re M., Ayanz J.M.S.M., Val E.P., Alvarado-Piqueras A., Fernández-Valeriano R., Blanco-Murcia J. (2025). Prevalence of bacteria involved in bovine respiratory disease in dairy heifers in Spain: Influence of environmental factors. Front. Vet. Sci..

[8] Jorritsma R., de Jong R., van den Hoven M., van Werven T. (2024). BRSV seroprevalence and associated risk factors on Dutch dairy farms. Vet. J..

[9] Klem T.B., Rimstad E., Stokstad M. (2014). Occurrence and phylogenetic analysis of bovine respiratory syncytial virus in outbreaks of respiratory disease in Norway. BMC Vet. Res..

[10] da Silva Barcelos L., Ford A.K., Frühauf M.I., Botton N.Y., Fischer G., Maggioli M.F. (2024). Interactions between bovine respiratory syncytial virus and cattle: Aspects of pathogenesis and immunity. Viruses.

[11] Milicevic V., Šolaja S., Gliši D., Ninković M., Milovanović B., Ðordević M., Ristevski S., Spasojević F., Dacić M. (2024). Bovine parainfluenza virus 3 and bovine respiratory syncytial virus: Dominant viral players in bovine respiratory disease complex among Serbian cattle. Animals.

[12] Bidokhti M.R.M., Tråvén M., Ohlson A., Zarnega B., Baule C., Belák S., Alenius S., Liu L. (2012). Phylogenetic analysis of bovine respiratory syncytial viruses from recent outbreaks in feedlot and dairy cattle herds. Arch. Virol..

[13] Jia S., Yaoa X., Yanga Y., Niua C., Zhao Y., Zhang X., Pan R., Jiang X., Xiaobo S., Qiao X. (2021). Isolation, identification, and phylogenetic analysis of subgroup III strain of bovine respiratory syncytial virus contributed to outbreak of acute respiratory disease among cattle in Northeast China. Virulence.

[14] Arns C.W., Campalans J., Costa S.C., Domingues H.G., D’Arce R.C., Almeida R.S., Coswig L.T. (2003). Characterization of bovine respiratory syncytial virus isolated in Brazil. Braz. J. Med. Biol. Res..

[15] Kaplan M., Özan E., Pekmez K., Çağırgan A.A., Arslan F. (2023). Molecular characterization of G and F protein genes of bovine respiratory syncytial virus detected from dead calves caused by severe respiratory syndrome: Emergence of novel mutations and their importance. Virus Dis..

[16] Valarcher J.-F., Schelcher F., Bourhy H. (2000). Evolution of bovine respiratory syncytial virus. J. Virol..

[17] Valentova V. (2003). The antigenic and genetic variability of bovine respiratory syncytial virus with emphasis on the G protein. Vet. Med-Czech.

[18] Zewde D., Gemeda G., Ashagrie T. (2022). Review on bovine respiratory syncytial virus characteristic, pathogenesis and control methods applied for the disease. Austin J. Vet. Sci. Anim. Husb..

[19] Anderson J., Do L.A.H., van Kasteren P.B., Licciardi P.V. (2024). The role of respiratory syncytial virus G protein in immune cell infection and pathogenesis. eBioMedicine.

[20] Valarcher J.-F., Taylor G. (2007). Bovine respiratory syncytial virus infection. Vet. Res..

[21] Yazici Z., Ozan E., Tamer C., Muftuoglu C., Barry G., Kurucay H.N., Elhag A.E., Cagirgan A.A., Gumusova S., Albayrak H. (2020). Circulation of indigenous bovine respiratory syncytial virus strains in Turkish cattle: The first isolation and molecular characterization. Animals.

[22] Struck A., Forster J., Ihorst G., Werchau H., König W., König B. (2004). Respiratory syncytial virus: G gene genotype and disease severity. Pediatr. Infect. Dis. J..

[23] Kumagai A., Kawauchi K., Andoh K., Hatama S. (2021). Sequence and unique phylogeny of G genes of bovine respiratory syncytial viruses circulating in Japan. J. Vet. Diagn. Investig..

[24] Aydin O., Yilmaz A., Turan N., Richt J.A., Yilmaz H. (2024). Molecular characterisation and antibody response to bovine respiratory syncytial virus in vaccinated and infected cattle in Turkey. Pathogens.

[25] Elvander M., Vilcek S., Baule C., Uttenthal A., Ballagi-Pordany A., Belak S. (1998). Genetic and antigenic analysis of the G attachment protein of bovine respiratory syncytial virus strains. J. Gen. Virol..

[26] Bertolotti L., Giammarioli M., Rosati S. (2017). Genetic characterization of bovine respiratory syncytial virus strains isolated in Italy: Evidence for the circulation of new divergent clades. J. Vet. Diagn. Investig..

[27] Virakul P., Suadsong S., Suwimonteerabutr J., Singlor J. (1997). Prevalence of infectious bovine rhinotracheitis (IBR), bovine viral diarrhea (BVD), parainfluenza-3 (PI-3) and bovine respiratory syncytial (BRS) viruses in Thai dairy farms. Thai J. Vet. Med..

[28] Saipinta D., Panyamongkol T., Chuammitri P., Suriyasathaporn W. (2022). Reduction in mortality of calves with bovine respiratory disease in detection with influenza C and D virus. Animals.

[29] Horwood P.F., Gravel J.L., Mahony T.J. (2008). Identification of two distinct bovine parainfluenza virus type 3 genotypes. J. Gen. Virol..

[30] Ravishankar C., Nandi S., Chander V., Mohapatra T.K. (2012). Glycoprotein C gene based molecular subtyping of a bovine herpes virus-1 isolate from Uttar Pradesh, India. Indian J. Virol..

[31] Tamura K., Stecher G., Kumar S. (2021). MEGA11: Molecular evolutionary genetics analysis version 11. Mol. Biol. Evol..

[32] Yaegashi G., Seimiya Y.M., Seki Y., Tsunemitsu H. (2005). Genetic and antigenic analyses of bovine respiratory syncytial virus detected in Japan. J. Vet. Med. Sci..

[33] Timurkan M.O., Aydin H., Sait A. (2019). Identification and molecular characterization of bovine parainfluenza virus-3 and bovine respiratory syncytial virus—First report from Turkey. J. Vet. Res..

[34] Zhou Y., Guo T., Zhao H., Hao Y. (2025). Epidemiological investigation and phylogenetic analysis of bovine respiratory disease complex in northern China. Vet. Med. Sci..

[35] Stine L.C., Hoppe D.K., Kelling C.L. (1997). Sequence conservation in the attachment glycoprotein and antigenic diversity among bovine respiratory syncytial virus isolates. Vet. Microbiol..

[36] Doreleijers J.F., Langedijk J.P., Hård K., Boelens R., Rullmann J.A., Schaaper W.M., van Oirschot J.T., Kaptein R. (1996). Solution structure of the immunodominant region of protein G of bovine respiratory syncytial virus. Biochemistry.

[37] Langedijk J.P., Meloen R.H., Taylor G., Furze J.M., van Oirschot J.T. (1997). Antigenic structure of the central conserved region of protein G of bovine respiratory syncytial virus. J. Virol..

[38] Furze J., Wertz G., Lerch R., Taylor G. (1994). Antigenic heterogeneity of the attachment protein of bovine respiratory syncytial virus. J. Gen. Virol..

[39] Furze J.M., Roberts S.R., Wertz G.W., Taylor G. (1997). Antigenically distinct G glycoproteins of BRSV strains share a high degree of genetic homogeneity. Virology.

[40] Prozzi D., Walravens K., Langedijk J.P., Daus F., Kramps J.A., Letesson J.J. (1997). Antigenic and molecular analyses of the variability of bovine respiratory syncytial virus G glycoprotein. J. Gen. Virol..

