# Supplementary material for: Molecular Characterization and Phylogenetic Analysis of Subgroup III Bovine Respiratory Syncytial Virus from a Dairy Outbreak in Thailand
Source: Vet Sci. 2026 Feb 26;13(3):220. doi: 10.3390/vetsci13030220 (PMC13030428; doi:10.3390/vetsci13030220)
Supplement: Supplementary file 1 [file vetsci-13-00220-s001.zip › vetsci-4147680-supplementary.pdf]

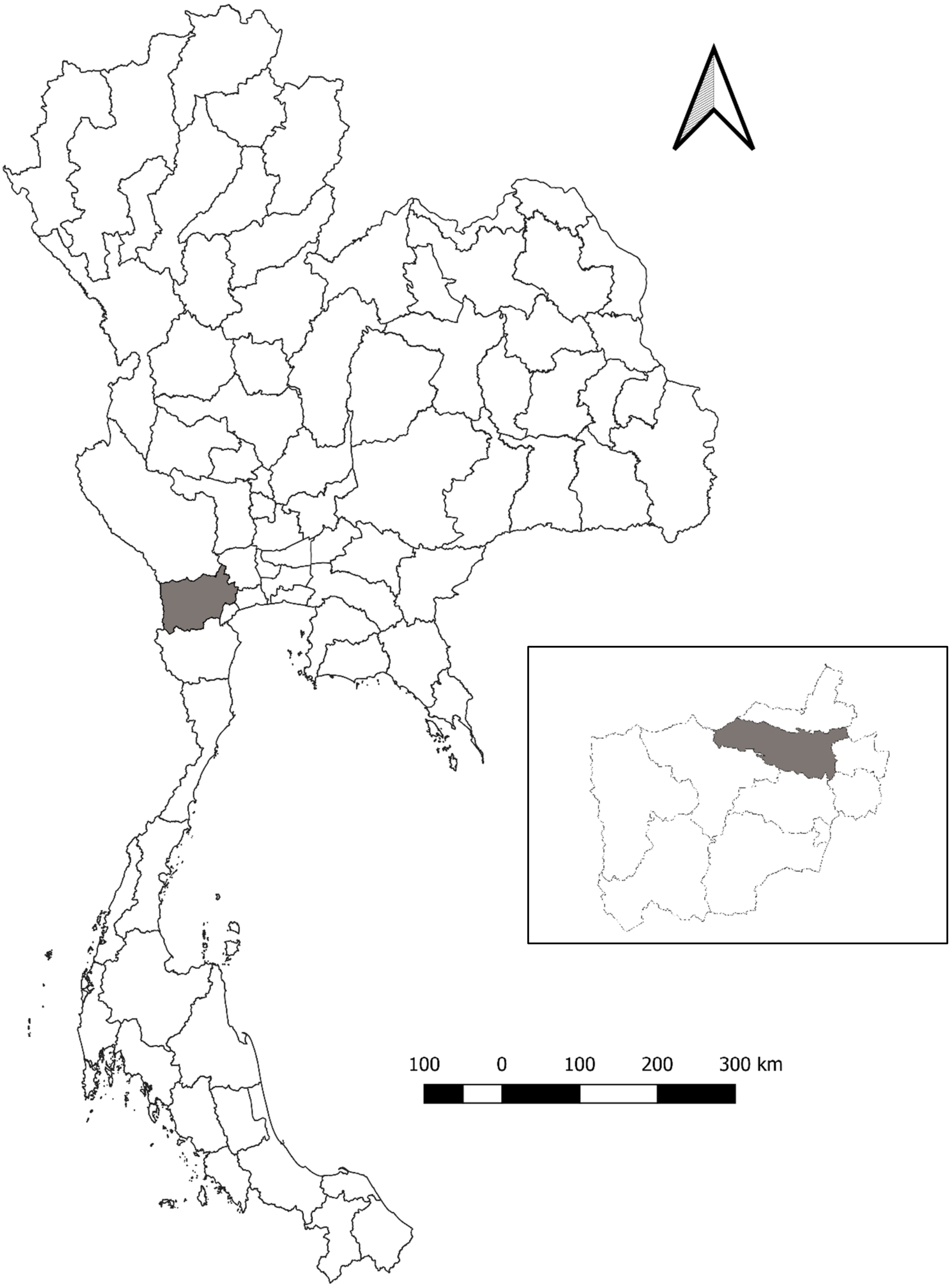

Supplementary Figure S1. Figure of Thailand map showing Ratchaburi Province in dark gray. The inset map expands the view of Ratchaburi, highlighting Photharam District in dark gray, where the samples used in this study were collected. The scale bar represents distances of 100, 200, and 300 km.

Supplementary Table S1. Clinical and animal information of specimens used for molecular detection and phylogenetic analysis of bovine respiratory syncytial virus (BRSV).

No.	Sample	Breed	Age (Year)	Lactation No.	Clinical sign
1	Lung	Holstein Friesian	5	3	pyrexia, depression, anorexia, panting, and moist lung sounds
2	Nasal swab	Holstein Friesian	8	6	pyrexia, depression, anorexia, panting, and moist lung sounds
3	Nasal swab	Holstein Friesian	5	3	pyrexia, depression, anorexia, panting, and dull lung sound, subcutaneous emphysema
4	Nasal swab	Holstein Friesian	3	1	pyrexia, depression, anorexia, panting, and moist lung sounds

Supplementary S1. The DNA extraction protocol of IndiMag Pathogen Kit by using IndiMag 48 machine.

1. Prepare 96-well plate and add reagents in column 2,3 and 4 following the table below

Column		Reagent	Volume (µL)
**1**	Lysate	Lysate*	745
**2**	Wash 1	Buffer AW 1	700
**3**	Wash 2	Buffer AW 2	700
**4**	Elution	Buffer AVE	100

*Lysate is a mixture of Buffer VXL + Buffer ACB + MagAttract Suspension G+ Proteinase K and the sample

2. Add 400 µL of Buffer ACB in the first column
3. Add 100 µL of Buffer VXL into first column
4. Add 20 µL of Proteinase K into first column
5. Add 25 µL of MagAttract Suspension G into first column
6. Add 200 of homogenized sample into the first column
7. Insert the prepared 96- well plate to the IndiMag 48 machine, choose "Pathogen" protocol and press "Start"
8. After the extraction was done, pipette the eluted from the fourth column to 1.5 mL microtube.
